# Promising Practices in Promotion of Healthy Weight at Small and Medium-Sized US Worksites

**Published:** 2008-09-15

**Authors:** Pamela Williams-Piehota, James Hersey, Jennifer Alexander, Karen Bandel Isenberg, Adrienne Rooks, Phillip B Sparling, Mary D Hill, Diane O Dunet

**Affiliations:** RTI International; RTI International, Research Triangle Park, North Carolina; RTI International, Research Triangle Park, North Carolina; RTI International, Research Triangle Park, North Carolina; RTI International, Research Triangle Park, North Carolina; Centers for Disease Control and Prevention, Atlanta, Georgia; Centers for Disease Control and Prevention, Atlanta, Georgia; Centers for Disease Control and Prevention, Atlanta, Georgia

## Abstract

**Introduction:**

We developed a new evaluation method to identify promising practices for promoting healthy weight among employees at small and medium-sized worksites.

**Methods:**

We used a structured rating and selection process to select 9 worksites with approximately 100 to 3,000 employees from a pool of worksites with health promotion programs reputed to be exemplary. A site visit over 2 sequential half-days at each site included interviews with senior management, program staff, vendors, and wellness committees; observation guided by a written environmental assessment; and structured review of program data on health outcomes of wellness program participants. The team corroborated findings from interviews, observations, and reviews of aggregate data on health outcomes of participants. Using the site visit reports, the project team and a separate panel of experts identified worksite health promotion practices that were promising, innovative, feasible to implement in a variety of settings, sustainable, and relevant for public health.

**Results:**

Innovative practices included peer coaching, wellness screening coupled with motivational interviewing and follow-up, free access to fitness facilities, and incentives such as days of paid leave for participation in wellness programs. Introduction of incentives was associated with higher participation rates. To build the business case for their programs, staff at several worksites used aggregate data on decreases in high blood pressure, serum cholesterol concentrations, and body weight in longitudinal samples of program participants.

**Conclusion:**

The evaluation method identified promising practices implemented at small and medium-sized worksites to promote healthy weight and related favorable health outcomes.

## Introduction

Obesity is among the leading causes of preventable death in the United States (1), and its prevalence has increased by 70% over the past decade (2). Medical expenditures attributable to overweight and obesity account for 9.1% of annual US medical expenditures, and these costs may be as high as $93 billion (3). Faced with increasing obesity-related diseases and associated costs, many employers are implementing a variety of health promotion programs: 46% of employers offer some type of physical activity program, 38% provide weight management programs, and 25% offer disease management programs that address obesity (4).

A limited number of studies of worksite health promotion programs demonstrate success in reducing obesity (5). A systematic review of 7 studies (5,6) found worksite programs that combined nutrition and physical activity were successful in encouraging initial weight loss, in the range of 4 to 26 pounds. However, studies with follow-up times longer than 6 months were generally less positive, which suggests that weight regain may be common (6). Furthermore, most worksite health promotion studies (5-16) have been conducted at large worksites (>5,000 employees), yet more than 70% of adults in the US workforce are employed in organizations with fewer than 5,000 employees (17). Consequently, we implemented a project to identify and assess promising health promotion practices in organizations we defined as small (<300) and medium-sized (300-5,000).

The evaluation project was not designed to establish firm conclusions about the effectiveness of specific practices; rather, we aimed to identify promising practices that merit additional, more rigorous study. We used a rapid evaluation method called Swift Worksite Assessment and Translation (SWAT), developed specifically to evaluate worksites one at a time (as opposed to comparing worksites to one another) by using predetermined criteria. We describe the process used to select 9 initial sites for SWAT assessments and present examples of practices that worksite health promotion experts deemed promising or innovative. We reflect on these examples and the observations we made at the 9 sites to stimulate thinking about worksite health promotion strategies in small and medium-sized worksites and potential areas for further research and evaluation.

## Methods

### Approach

The SWAT evaluation method we used for this project was developed to rapidly assess worksite strategies to help employees attain and maintain a healthy body weight. A companion article in this issue of Preventing Chronic Disease (18) provides an account of the SWAT development process, its key features and operational steps, and a brief summary of an independent evaluation conducted to assess the SWAT method itself and its effectiveness as an evaluation approach. For the SWAT assessments of the 9 worksite health promotion programs reported here, we used key operational definitions developed for SWAT (Appendix). These definitions were also used in 2005 as a working framework for a systematic review of the “gray” literature that was being conducted to supplement a Task Force on Community Preventive Services literature review in Guide to Community Preventive Services (6). We defined a promising worksite practice as an innovative worksite strategy supported by field-based, aggregate data showing 1) no weight gain and a positive change in at least 1 related behavioral marker (eg, physical activity or dietary pattern) or biomedical marker (eg, blood pressure or serum cholesterol concentrations) in employees who had normal weight or 2) sustained weight loss in employees who were overweight or obese.

### Sample selection

Table 1 shows the steps completed to identify and select worksites. Potentially promising practices were suggested by a panel of nationally recognized health promotion experts engaged as consultants to develop the SWAT method. We compiled a list of US worksites that used these practices and supplemented it with the names of worksites found in Internet searches of sources, such as the Wellness Councils of America, Partnership for Prevention, and the National Business Group on Health. Finally, wellness professionals nominated other programs in response to announcements posted on health- and business-related listservs.

We sent a brief description of the project and a personal invitation to participate to 41 small and medium-sized worksites that met the inclusion criteria defined by the SWAT method. Sixteen worksites responded, and we conducted brief telephone interviews to obtain information on the main components of their health promotion programs. We then prepared summaries of the telephone interviews and used coding to omit employer names and locations.

The project team reviewed each of the 16 worksite health promotion program summaries and used a structured rating process to score each program to determine whether to recommend the site for a visit. Concurrent with this activity, 3 senior CDC staff members with expertise in worksite health promotion acting as an expert panel also scored the programs. If additional information was necessary to complete the ratings, we conducted brief follow-up calls to obtain such information.

Funding was available to conduct up to 9 initial SWAT assessments; ideally, these would cover a wide variety of workplace settings. The project team selected the 9 worksites with the highest ratings based on a combined score of the project team and the CDC expert panel, after confirming that the 9 sites provided a range of organizational types and sizes. As shown in Table 2, the worksites included manufacturing, construction, health care, higher education, and government organizations, and they varied in workforce size from approximately 100 to 3,000 employees.

### Measures

A 1-day site visit (typically conducted as 2 consecutive half-day visits) at each worksite allowed the project team to assess the worksite health promotion program and to make observations firsthand. Structured topic guides were used for key informant interviews with 1) the health promotion director and staff responsible for delivering the intervention (22 questions, approximately 2 hours for the program director and 1 hour for program staff), 2) the data collector or analyst for the program (27 questions, approximately 1 hour), and 3) the human resources director, upper-level manager, or chief executive officer, that is, upper-level decision makers who supported or funded the program (11 questions, approximately one-half hour). At some sites, we had discussions with employee wellness advisory committees (11 questions, approximately 1 hour).

During the interviews, we gathered data on worksite characteristics, including the size of workforce, the type of jobs or industry represented at the site, and employee sociodemographic characteristics. The structured topic guides included questions about the staffing for the health promotion/healthy weight practice, program resources, and operating costs. We also asked questions about the health promotion program objectives, activities, innovativeness, and factors that contribute to successful implementation and sustainability. The guides included a series of questions about program participation, including eligibility requirements, the percentage of eligible employees who participate, and whether specific groups are targeted at the worksite. To track program participation, we asked specific questions about which types of employees actually participate, what activities they participate in, the program-related variables that are measured and their frequency, and the program completion rates. We also asked about results from the program, including any changes in the worksite environment or policies or the weight and health-related behaviors of employees (eg, diet, physical activity). Finally, we inquired about sources of support for healthy weight and for the program, including senior-level support and the community environment.

Site visitors were given a guided tour of the worksite, or a portion of it, to conduct a written environmental assessment of such features as stairwells, cafeteria or lunchroom facilities, fitness areas, products in vending machines, and other environmental features. The assessment followed a structured checklist adapted from the *Checklist of Health Promotion Environments at Worksites* (94 questions, approximately 1.5 hours) (19).

We also used a structured form to guide our review of program documents that provided aggregate data on the health practices and health status of program participants. Interview guides for key informants and the environmental assessment checklist can be found at http://www.cdc.gov/nccdphp/dnpa/hwi/.

The project was designed for rapid assessment so that promising practices could be identified and, potentially, evaluated more rigorously. In keeping with privacy rules, we did not collect individual-level data or analyze it to verify the accuracy of the aggregate data on program participation, behavior, or health status shared by employers. We did, however, independently check that the interpretation of aggregate data on program participation or health status was described in our site visit reports in a manner that was consistent with accepted evaluation standards (20-23). Typically, this meant that our site visit report described the limitations that should be placed on interpretation of aggregate changes in health status, such as the possibility that results may have been affected by self-selection (ie, employees who participate in health promotion programs may be more motivated than nonparticipants), differential attrition (ie, employees who are making progress toward health goals may be more likely to stay in a health promotion program than those who are not as successful), or secular trends (ie, other changes in the community).

### Analysis

After each of the 9 site visits, we summarized written interview notes, the data inventory checklist and notes, and the environmental checklist completed during the site visit. Teams of 2 evaluators who made the site visits collaborated to synthesize the information in a descriptive report of each worksite program. In summarizing each site, we sought corroboration of evidence among sources, including consistency among respondents. We also examined consistency among respondent self-reporting, information in written documents, and observations made by site visitors. The site visit reports also provided detail on the contexts of the worksite and the community, program components, program participation, and evidence for program effects on weight and health. Furthermore, the reports described the strengths and limitations of aggregate data on participation and health status provided by the worksites. Draft site visit reports were then sent to each site to verify facts and add relevant information. As with the site selection process, the project team and the CDC expert panel used a structured rating process to score sites based on criteria defined in the SWAT method. Scoring criteria included feasibility of practice implementation in a variety of worksite settings; sustainability of the practice and of its apparent health effects; relevance for public health; and cost. Raters provided a final overall summary rating for each site.

As a final step, the 3-member expert panel was asked to identify particular strategies or practices they considered to be promising or innovative on the basis of their knowledge of worksite health promotion. The following results reflect the expert panel's conclusions.

## Results

Several innovative practices were identified by the expert panel. Table 3 gives examples of how worksites implemented strategies that experts deemed notable. Our synthesis of the expert panel's conclusions for the 9 worksites we visited reinforce the view that successful worksite strategies encompass individual, environmental, and organizational factors.

### Types of strategies

**Individual level**

Experts observed that several worksites offered high-tech wellness screening procedures and Web-based data-management systems, along with personal counseling about results. For example, participants could get periodic counseling, health risk assessments (HRAs), and rapid measurements of blood pressure, serum cholesterol concentrations (ie, fingerstick testing), and obesity (ie, body mass index [BMI], waist circumference, and skinfold testing). These assessments were supported by Web-based data-management systems that allowed the health counselor to access screening information so participants could monitor their progress.

Another feature of the programs identified by experts was the "high-touch" component, such as motivational interviewing, regular follow-up, and peer support. At a construction company, for example, participants in the employee wellness program met one-on-one with a health educator who was trained in motivational interviewing. The educator helped employees complete an HRA, review personal health risks, and set short- and long-term goals. All participants received a printout of their health risks, total health risk score, personal goal, and resources for health information related to that goal. Participants were required to meet with a health educator for 30-minute follow-up sessions several times throughout the year, as determined by their health risk category. At a manufacturing company, an occupational health nurse conducted personal monthly health check-ins with employees to discuss their progress toward self-selected health goals.

**Environmental level**

The expert panel observed that a number of the worksites had taken action to provide increased access to programs to boost participation. For instance, a community college provided free access for staff to use a fitness facility because they thought having staff exercise beside students helped to further the school's sense of community. One program increased the hours and locations in which health assessments and screening were provided, and another negotiated use of a fitness facility in a nearby hotel, to provide access to staff in an offsite location.

**Organizational level**

Experts identified strong support from senior management as an important feature, and nearly all (8 of 9) programs we visited were deemed to have such support. For example, senior management spearheaded and started the program; directly encouraged employees to participate, participated in program activities, held organization-wide meetings to discuss the concept of wellness and to present program details, and said the wellness program was important for a healthy workforce. Many staff members in these programs used evaluation results to help sustain the interest of senior managers.

Several programs encouraged mobilization of a health-promoting worksite culture. For example, a manufacturing company had visible encouragement and support from senior management. The program was spearheaded by the company president, who showed up at 8 am for weight-loss team weigh-ins. The community college program offered employee-led walking and jazzercise classes. Walking clubs were established to facilitate a primary goal of the community health care program — increasing the number of walking steps taken. Employees were trained to lead these clubs, and for doing so they earned activity points toward program monetary incentives.

Promising practices related to wellness often involved changes in organizational policy. For instance, wellness was incorporated into the mission statement of the metal-finishing company. Staff at one worksite wrote requirements for nutritious foods into their criteria for selecting the vendor that operated the cafeteria. The cafeteria manager reported that with the introduction of healthier food choices, more people started using the cafeteria. The construction company gave employees notice that within 1 year, all construction sites would be tobacco-free and offered free counseling for smoking cessation and coverage for nicotine replacement therapy.

Experts noted as innovative the use of financial incentives to encourage participation in programs. At the metal-finishing company, for example, wellness goals were tied to annual performance reviews. All employees were evaluated on safety, performance, and attendance, including wellness activities. Employees met expectations if they attended each quarterly health screening and exceeded expectations if they attended all quarterly screenings *and* met their wellness goals. Most managers were placed in incentive compensation plans, with 10% of their bonus directly related to achieving their wellness goals. The small manufacturing company changed the structure of its benefits to promote them as incentives for program participation, so that the company's previous contribution toward health insurance deductibles was instead tied to attainment of specific health goals (eg, a benefit for BMI ≤30 kg/m^2^).

To further extend the social support component of their programs, several worksites encouraged participation of spouses of employees. In the aviation-support company, the wellness program was available to all employees and their spouses. If both participated in the annual health screening, they received a 10% discount on the health insurance premium. The construction company contributed approximately $600 per person to married employees toward their deductible only if both the employee and the spouse participated in the program. Providing for spousal participation was consistent with reinforcing employee participation. In most (approximately 60%) of the programs with participation rates of 70% or higher, spouses were allowed to participate in all program components.

The expert panel members were also impressed by the ability of these small and medium-sized worksites to rapidly implement changes. For example, the small manufacturing company modified its program from a team competition, to an individual weight-loss program, to a screening program with financial incentives for meeting specific wellness goals and the intention to make the goals increasingly strict each year. The health care provider changed its incentive for participating in its wellness program from a $200 flex credit for health insurance coverage to a debit card allowing participants to earn up to $190 per year for participating in the program. Money earned could be spent anywhere, not just on health insurance. Then, the program added a disincentive; employees and spouses who were on the company's health plan but did not participate in the wellness program had to pay a surcharge on their health insurance premium of approximately $30 per pay period ($770 per year).

### Evaluation

Program staff at worksites used evaluation of participation and health effects to guide program development and build the business case to sustain the programs in terms of the resulting savings in health care costs. One method they used was to build in measures and procedures to assess and refine their programs.

**Participation**

To refine program outreach and offerings, staff at some sites used data on participation as an early indicator of success. For example, a city government instituted an annual planning process with its wellness committee. On the basis of participation rates in various departments, coupled with responses to a survey of employees about program interests, management introduced the incentive of paid leave for up to 2 days for completing the HRA, having an annual health screening, and earning points through participation in ongoing wellness programs. The worksite staff reported that this change increased participation rates among men from 24% to 60% the year after this change was implemented. In addition, program staff frequently cited aggregate data on participant health outcomes to help build the business case for the program.

**Health effects**

*Behavior*. Of the 9 wellness programs, 5 assessed changes in aggregate employee health behavior from HRAs. For instance, the health care provider shared aggregate data indicating that the percentage of participants who reported "exercising for at least 20 minutes" less than once per week decreased from 30% (202/679) in 2002 to 16% (108/679) in 2005. Because worksite program staff understood the limited reliability of self-reported behavioral data, they placed greater emphasis on measurement of health risk through more objective measures, such as biometric data (eg, measured body weight, blood pressure, serum cholesterol concentrations).

*Weight loss, healthy weight, and health outcomes.* Five of the 9 programs assessed longitudinal data on weight status, although the metrics varied. For example, program staff in a health care setting reported that 35% (122/353) of overweight participants (defined by the program as BMI >27.5 kg/m^2^) lost more than 4 pounds over 12 or more months. A construction company shared a report of aggregate data indicating that 16% of employee participants decreased their risk for being overweight (BMI >27.5 kg/m^2^) over a 2-year period compared with baseline. Program staff from a community health setting reported an average weight loss of 6 pounds per participant (N = 90) over a 1-year period.

Five of the worksites had collected repeated cross-sectional or longitudinal data on blood pressure or serum cholesterol concentrations in aggregate data from health assessments. For example, the metal-finishing company reported data that showed that the percentage of participants with hypertension stages 1 and 2 (blood pressure ≥140/90 mm Hg) decreased from 17.4% in the first quarter of 2005 to 12.0% in the fourth quarter of 2005. The community college shared repeated, cross-sectional aggregate data showing reduction in high serum cholesterol concentrations from 16% of participants in 2003 to 10% in 2005. The construction company's aggregate data indicated that, over a 2-year period, in a longitudinal sample of approximately 2,000 employees, 20% decreased their risk for high cholesterol. In the absence of data from a comparison group, knowing how much of this change was attributable to program participation is difficult. Nonetheless, data on aggregate changes in physical health status were used to build the business case for the wellness program.

To further build the business case for health promotion, several worksite programs also reported to state or regional worksite associations on changes in health care costs or on reduction in the rate of increase in health care costs since the wellness program was established. At worksites where this was done, the programs appeared to have more than paid for themselves, although determining how much of this change can be attributed to program participation is difficult. However, staff at one worksite used establishment of a wellness program as a rationale to convince their insurer to change the organization's insurance rating status, resulting in lower health insurance premiums.

## Discussion

The goal of this evaluation was to identify promising practices for promoting healthy weight in small to medium-sized worksites. We used the SWAT rapid-assessment approach for evaluation — a middle-ground approach between informal assessment and resource-intensive rigorous research (18). Although we were unable to definitively evaluate the effects of specific practices on health status, we did identify a number of innovative or notable worksite practices deemed promising (Table 3).

Considerable similarity exists between the small and medium-sized worksites we studied and larger worksites described in other studies; for example, both offer a combination of nutrition and physical activity programs for preventing and controlling overweight and obesity, as recommended by the Task Force on Community Preventive Services (6). Several programs in our study offered nutrition education in combination with onsite exercise facilities or physical activity programs. A previous study (24) reported that worksite programs aimed at increasing consumption of fruits and vegetables were more effective if they 1) were based on social ecological approaches (25), 2) encouraged employee participation in program planning and implementation, 3) addressed multiple risk factors related to behavioral change, and 4) included employees’ broader social context. We did not assess the effectiveness of our programs in increasing fruit and vegetable consumption. However, many of the programs we evaluated in this study implemented strategies at multiple levels of social ecological change, involved employees in program planning through employee wellness committees and in implementation through activities such as employee-led walking clubs and peer coaching, addressed multiple behavioral risk factors, and included employees’ spouses. This study is useful because it considered worksites smaller than those previously studied and identified practices that merit rigorous evaluation in these types of worksites.

The 9 worksite programs we visited were nominated as having exemplary programs, and they were chosen because they showed promise. Hence, we caution against trying to generalize these findings to other worksites. Indeed, this project was not designed to provide generalizable findings but rather to identify promising practices that might merit more rigorous evaluation.

Furthermore, the aggregate quantitative data on behavior and health status that the worksites shared with us had several limitations. Worksite staff tended to gather data on self-reported changes in behavior. Thus, it was difficult to know whether reported changes were associated with social desirability or with greater attention to and knowledge about the behaviors. Most worksites recorded measured (not self-reported) height and weight, yet change in weight status over time was not always analyzed and reported. Only a few programs reported longitudinal data, and in almost no cases were data available from comparison groups. The CDC expert panel members expressed a strong desire for more data or more rigorously analyzed data on program effectiveness, to help them better determine whether a practice was promising. They also noted the need for longitudinal data to demonstrate changes in health behavior or maintenance of weight loss.

Recognizing the subjectivity of interview and observational data, we took several actions to increase our confidence in our findings. First, we used a variety of data sources to corroborate evidence. For example, worksites' aggregate program data on health outcomes was compared with the information we collected from key informant interviews and the environmental assessment conducted during the site visits. Next, we provided worksite program staff a draft site visit report and asked them to verify its accuracy. To assess the rating process of the experts who read the site visit reports and identified promising practices, the project team completed the same rating process and compared results for consistency.

This evaluation project identified promising practices implemented at small and medium-sized worksites to promote healthy weight and related favorable health outcomes. Practices that appeared promising for small to medium-sized worksites included periodic health assessments tied to personal feedback and motivational interviewing, peer coaching, use of an occupational health nurse to check in monthly with employees, and changes in and promotion of benefits as incentives for program participation. This report suggests that more rigorous studies, such as randomized controlled trials, are merited to assess more thoroughly the effect of specific innovative and promising health promotion practices on health outcomes and to investigate whether these strategies could work for companies with fewer than 100 employees.

## Figures and Tables

**Table 1:** Identification and Selection of US Worksites for Evaluation Using the Swift Worksite Assessment and Translation Method, 2005-2006

**Step in Process**	**No. of Sites**	**Description of Step**
**1**	41	Potential sites identified through Internet searches, nominations from health promotion experts, award programs, and recommendations by colleagues. Personal letters sent to sites inviting participation in the project.
**2**	16	Sites respond to the initial invitation.
**3**	16	RTI conducts brief telephone interviews with responding sites to obtain general information on worksite health promotion program and self-evaluation activities, especially with regard to practices related to healthy body weight.
**4**	14	CDC expert panel members review 2-page summaries of each responding employer and rate worksites according to study criteria. Of the 16 potential sites identified for site visits, 4 were strongly recommended and 2 were not recommended. Experts request additional information on 8 sites.
**5**	8	RTI conducts follow-up telephone interviews to obtain requested data for further consideration by expert raters.
**6**	9	RTI/CDC project team selects 9 sites that were scored highest and confirms diversity of organizational type and size.

Abbreviations: RTI, RTI International; CDC, Centers for Disease Control and Prevention.

**Table 2:** Overview of Programs to Promote Healthy Weight Among Employees in Small and Medium-Sized US Worksites Evaluated With the Swift Worksite Assessment and Translation Method, 2005-2006

Site Description	**Selected Program Components**
**Industrial worksites**	
**Manufacturing company**	Annual wellness screening program, including biometric measures Increased company reimbursement for the copay portion of health insurance as an incentive for participation in screening program Occupational nurse who meets with every employee once per month to discuss progress toward employee's self-selected health goals Team weight-management competition, followed by an individualized weight-maintenance program
Size: 115 employees	
Program operation: 3 years	
**Metal-finishing company**	Employee wellness goal in company's mission statement Expected participation in quarterly health screenings and setting of wellness goal Wellness program structured with gold-, silver-, and bronze-medal rankings to recognize incremental levels of health behaviors Free annual mountain hike in Colorado with chief executive officer and senior management for employees who meet highest levels of safety and health goals
Size: 450 employees	
Program operation: 15 years	
**Aviation-support company**	Free annual onsite biometric screening and health risk assessments for employees and spouses Free access to onsite fitness center One-on-one consultations and 3-month follow-ups with wellness staff
Size: 1,800 employees	
Program operation: 8 years	
**Construction company**	One-on-one meetings with health educator for employees at all job sites to complete HRAs, review health risks, and set short- and long-term goals Ongoing counseling based on motivational interviewing to bring about behavior change Onsite daily mandatory stretching program that is linked to occupational safety for all employees
Size: 2,000 employees	
Program operation: 3 years	
**Governmental/Academic Worksites**	
**Community college**	Free HRAs and biometric health screenings with immediate counseling every 6 months for full-time employees and their spouses Free access to college campus fitness center1 hour of paid leave per week for staff to participate in wellness activities Inclusion of healthy food requirements in selection criteria for cafeteria vendor
Size: 375 employees	
Program operation: 3 years	
**City government**	Free annual onsite health risk assessments and health screenings A variety of physical activity and nutrition activities throughout year Introduction of additional vacation time as incentive for participation in health promotion program Close link with health cost management consultant Involvement of wellness committee in the annual planning process
Size: 240 employees	
Program operation: 10 years	
**Research facility**	Fully staffed onsite fitness and health center Year-long weight-management program with individual and team competitions for employees, families, and retirees One-on-one nutrition counseling available with onsite registered dietitian
Size: 3,200 employees	
Program operation: 23 years	
**Health Care Worksites**	
**Community health provider**	Employee wellness program that is outgrowth of health services provided to patients Network of volunteer peer health coaches
Size: 425 employees	
Program operation: 1 year	
**Health care provider**	Personal health coaching sessions every 3 months with health educator Strong financial incentives (more than $900/year) tied to completion of 3 coaching sessions and 3 educational units completed throughout year
Size: 3,100 employees	
Program operation: 4 years	

Abbreviation: HRA, health risk appraisal.

**Table 3:** Promising Strategies to Promote Healthy Weight Among Employees in Small and Medium-Sized US Worksites Evaluated With the Swift Worksite Assessment and Translation Method, 2005-2006^a^

**Individual Level**
Periodic health assessments tied to personalized feedback and individual coaching and motivational interviewing Monthly walk-through of entire worksite by occupational health nurse (vendor) during which she visits with all employees and is available to discuss health concerns The use of peer coaching to deliver the program, whereby employees are trained as health coaches and meet monthly with participants to collect program activity points and measure progress (eg, changes in weight and blood pressure) toward achieving positive health outcomes Strong linkage of wellness program with worker safety, including group stretching to promote model of an "industrial athlete" Health coaches who travel among a company's worksites to meet with employees
**Environmental Level**
Strong support from wellness committee for establishing culture of wellness Inclusion of healthy food in criteria for selecting cafeteria vendor Free access to onsite physical activity facilities Provision of bicycles for travel between buildings
**Organizational Level**
Incentive of paid day of leave to encourage program participation Reallocation of existing benefits to provide incentives for participating in screening activities and attaining wellness goals Strong financial disincentives for employees and spouses with health insurance through the company who do not participate in wellness program Integration of wellness goals into work performance expectations

a As identified by an expert panel from the Centers for Disease Control and Prevention.

## Appendix. Operational Definitions That Guided the Swift Worksite Assessment and Translation (SWAT) Project

**Healthy weight (in adults): **A body weight that falls within the healthy body mass index range of 18.5-24.9 kg/m^2^.

**Practice:** A strategy, intervention, or policy designed to affect individual health positively. For this project, practices either focused on healthy weight or indirectly benefited healthy weight. For example, physical activity strategies that are not weight-focused are included, but smoking cessation programs are not (unless they include a weight control component).

**Worksite practices:** Health promotion strategies delivered by an employer to employees at a designated physical workplace. These include a wide array of strategies and can be implemented on single or multiple levels (eg, individual, organization, community).

**Innovation: **A new approach or adaptation in the promotion of healthy weight among employees. Established practices that have been evaluated formally were not the focus of this project. New components, re-inventions, or adaptations of evidence-based practices were considered — for example, practices that have been shown to be effective with other behaviors such as smoking or that target hard-to-reach employees.

**Positive (weight-related) outcome:** Employee group data that indicate either weight loss or no weight gain *and* include a positive change in at least 1 related behavioral marker (eg, physical activity, dietary patterns) or biomedical marker (eg, body composition, blood pressure, blood lipids, blood glucose).
